# Auditing in the Irish Casemix budget models

**DOI:** 10.1186/1472-6963-11-S1-A1

**Published:** 2011-10-19

**Authors:** MD O'Connor, E Burke, E Gallagher, F Bane, J Johnson

**Affiliations:** 1 HSE - National Casemix Programme, Millennium Park, Naas, Co. Kildare, Ireland

## Introduction

Casemix in Ireland is run on a retrospective basis. Budget adjustments are made that relate to costs and activity for the calendar year two years previously. This is presently changing to a prospective funding model which will be informed by a Patient Level Costing project currently in progress.

The Irish Casemix budget models are subject to a rigorous audit process. A unique aspect of Casemix in Ireland is that it is budget neutral. The effect of this is that the performance of one hospital affects all of its peer hospitals. To ensure confidence in the process, there is a detailed audit done on the costs and activity of each hospital. Cost audits have been part of the annual Casemix process since Casemix was introduced in Ireland in 1991. In recent years, the National Casemix Programme (NCP) has put an increased focus on activity auditing.

The staff in the NCP have direct access to and regular communication with costing and coding staff in each hospital. This access is essential in enabling a thorough audit process.

## **Methods**

### **Costing**

The NCP has a number of large, standardised Excel files – designed in house – in which each hospital must return its costs broken down by specialty. The completion of these files must be in accordance with the Costing Manual, which is updated annually. A detailed review of each file is conducted against the previous year. This results in a list of queries being sent to the costing staff in each hospital, with a particular focus on costs being allocated to areas outside Casemix, or where exposure to Casemix is low. This is repeated until the process is concluded. During this process, comparisons between hospitals are conducted to ensure a consistent approach is being applied.

### **Activity**

Activity is returned by each individual hospital in a monthly download. A monthly set of audit files is compiled by NCP statisticians to show hospital, MDC, and DRG data from a wide range of perspectives. These files allow top-down systematic and ad hoc interrogations of the data to be made. From the files, detailed annual queries to which hospitals must respond are drawn up on DRGs where:

1. There is a significant increase/decrease in DRG activity and value.

2. There is a significant increase/decrease in activity and value across an Australian Refined Diagnosis Related Group system (AR-DRG).

3. There is a trend towards more/less complexity within an AR-DRG.

There should be a direct relationship between changes in activity and changes in cost. Unexplained increases in activity, and monetary value related to these increases, can be targeted for on-site auditing and, if necessary, amended or excluded. The NCP employs a ‘Dampening’ principle which allows for the removal of unexplained increases in activity; as well, there is the addition of activity where significant decreases threaten a hospital’s funding base.

## **Results**

1. Detailed cost-review queries are sent annually to nominated staff at each hospital. Responses and further queries are exchanged until this process is finalised.

2. On-site costing audits are carried out if issues remain.

3. The suite of audit files allows a top-down audit of hospital activity and direct attention toward areas requiring further analysis.

4. The activity audit files sent to hospitals focus on possible cases of DRG creep. These files bring attention to activity increases or decreases which will have a positive or negative impact on Casemix performance.

5. The costing file allows analysis of cost per case to ensure that it is consistent and reasonable in all hospitals. This improves the quality of costs in the budget models.

6. The ‘Dampening’ rule ensures that unexplained increases or decreases in activity are removed. This results in relative consistency of funding for hospitals year on year.

## **Conclusions**

1. A credible Casemix model requires a high degree of visible audit.

2. The standardised file structure supported by a detailed Costing Manual allows confidence in how costs are reported to the NCP.

3. The cost audit process ensures that reliable costs are entered in the budget models.

4. The activity audit process focuses attention on areas of activity which will have a positive or negative impact on Casemix performance.

5. Further resources are required to expand the audit work carried out, particularly with the results from the Patient Level Costing project and the shift to Prospective Funding.

My presentation will display the approach taken by the Irish NCP to ensure the integrity of the budget models.

